# Age dependent differences in the kinetics of γδ T cells after influenza vaccination

**DOI:** 10.1371/journal.pone.0181161

**Published:** 2017-07-11

**Authors:** Ulrik Stervbo, Dominika Pohlmann, Udo Baron, Cecilia Bozzetti, Karsten Jürchott, Julia Nora Mälzer, Mikalai Nienen, Sven Olek, Toralf Roch, Axel Ronald Schulz, Sarah Warth, Avidan Neumann, Andreas Thiel, Andreas Grützkau, Nina Babel

**Affiliations:** 1 Berlin-Brandenburg Center for Regenerative Therapies, Charité –Universitätsmedizin Berlin, Augustenburger Platz 1, Berlin, Germany; 2 Center for Translational Medicine, Medical Clinic I, Marien Hospital Herne, University Hospital of the Ruhr-University Bochum, Hölkeskampring 40, Herne, Germany; 3 Epiontis GmbH, Rudower Chaussee 29, Berlin, Germany; 4 Institute of Biomaterial Science and Berlin-Brandenburg Center for Regenerative Therapies, Helmholtz-Zentrum Geesthacht, Centre for Materials and Coastal Research, Kantstraße 55, Teltow, Germany; 5 Deutsches Rheuma-Forschungszentrum Berlin–a Leibniz Institute, Charitéplatz 1, Berlin, Germany; Monash University, Australia, AUSTRALIA

## Abstract

Immunosenescence is a hallmark of the aging immune system and is considered the main cause of a reduced vaccine efficacy in the elderly. Although γδ T cells can become activated by recombinant influenza hemagglutinin, their age-related immunocompetence during a virus-induced immune response has so far not been investigated. In this study we evaluate the kinetics of γδ T cells after vaccination with the trivalent 2011/2012 northern hemisphere seasonal influenza vaccine. We applied multi-parametric flow cytometry to a cohort of 21 young (19–30 years) and 23 elderly (53–67 years) healthy individuals. Activated and proliferating γδ T cells, as identified by CD38 and Ki67 expression, were quantified on the days 0, 3, 7, 10, 14, 17, and 21. We observed a significantly lower number of activated and proliferating γδ T cells at baseline and following vaccination in elderly as compared to young individuals. The kinetics changes of activated γδ T cells were much stronger in the young, while corresponding changes in the elderly occurred slower. In addition, we observed an association between day 21 HAI titers of influenza A and the frequencies of Ki67^+^ γδ T cells at day 7 in the young. In conclusion, aging induces alterations of the γδ T cell response that might have negative implications for vaccination efficacy.

## Introduction

The seasonal influenza virus accounts for thousands of deaths and hospitalization of elderly in industrialized nations [1]. These numbers are likely to rise as life expectancy increases, resulting in an increased burden on the health care systems. The responsiveness of the immune system decreases with age thus diminishing the vaccine efficacy [2]. This in turn makes the weakened more susceptible to fatal infections.

γδ T cells intersect the innate and adaptive arms of the immune system [3]. The T cell receptor (TCR) of γδ T cells is, similar to that on αβ T cells, generated by a random combination of various gene-segments. Although the potential repertoire of γδ T cells is much larger than for αβ T cells, the actual diversity is much more restricted [4]. γδ T cells constitute about 1–10% of all CD3^+^ T cells in humans and are, according to their TCR expression, broadly divided into two groups: The epithelium associated Vδ1^+^ and Vδ3^+^ and the circulation associated Vδ2^+^, which constitutes 50–90% of γδ T cell in peripheral blood [5].

Similar to the cells of the innate immune system, γδ T cells can respond rapidly upon activation through pre-programmed release of particular cytokines including interferon (IFN)-γ, interleukin (IL)-4, or IL-17 [6]. The cells also have cytotoxic properties mediated by granzymes and the death receptor ligands FasL and TRAIL [7]. The adaptive properties of γδ T cells are found in their ability for clonal expansion after antigen-specific priming [6]. The cells further possess memory akin to other adaptive immune cells [8,9] and have been suggested to play a role in age related alterations of the immune response [10].

A range of antigens recognized by γδ T cells has been identified [4]. Among these are small peptides, membrane anchored proteins, phospholipids, prenyl pyrophosphates, and sulfatides. Soluble proteins, such as tetanus toxoid and heat shock proteins, have also been demonstrated to induce a γδTCR-dependent T cell response [5,11]. The recognition of soluble proteins might occur independently of MHC and other antigen presenting molecules [12].

CD38 is upregulated on γδ T cells after activation [13]. This is similar to αβ T cells, where several substrates for CD38 are known to regulate the functionality of the cells [14,15]. The proliferation marker Ki67 is well established as it is expressed in all phases of the cell cycle but the G0 phase [16]. The exhaustion marker PD-1 is also expressed on γδ T cells upon activation [17], but it is not yet clear if PD-1 signaling can effectively dampen the response of γδ T cells [18].

Human γδ T cells have also been found to act as professional antigen presenting cells (APCs) where they efficiently take up and present soluble antigen [19–22]. The role as APC might be an evolutionary conserved feature of γδ T cells [23]. In particular, the cells were shown to become activated by recombinant influenza hemagglutinin and present peptides derived from influenza virus particles to CD4^+^ and CD8^+^ αβ T cells [22,24]. Activated γδ T cells have been shown to home to the lymph nodes by the expression of CCR7 and to the gut by expression of αEβ7 [14,25]. In addition to their potential role as APCs, γδ T cells can also assist in maturation of dendritic cells (DCs) and can further help B cells into becoming antibody-producing plasma cells by secretion of cytokines [26].

γδ T cells play an important part in clearing virus infections [27]. This is achieved by direct killing of EBV, CMV, and HSV infected cells [28,29], or by non-cytolytic inhibition of HSV or SIV replication through secretion of IFN-γ [30,31]. The Vγ9Vδ2 γδ T cells can control infection by several strains of influenza virus, such as the pandemic H1N1, and the avian H5N1 and H9N2 viruses ex vivo [32–34].

The exact mode of interaction between the γδ T cells and the influenza virus is not fully understood. However, it has been suggested that γδ T cells are activated by the influenza hemagglutinin through binding to sialic receptors [24]. The anti-viral function of the γδ T cells relies on receptor mediated apoptosis induction through TRAIL or FasL [35].

Since hemagglutinin is a primary component of the seasonal influenza vaccine [36], it is possible that γδ T cells become activated following vaccination. We therefore asked how γδ T cells might affect vaccine efficacy. To this end, circulating γδ T cells were analyzed in young and elderly at day 0, 3, 7, 10, 14, 17, and 21 after vaccination with the trivalent 2011/2012 northern hemisphere seasonal influenza vaccine [37]. We focused on activated and proliferating γδ T cells as defined by the expression of CD38 or Ki67. We found that the elderly had significantly lower cell numbers in the days following the vaccination and that the younger had larger fluctuation in the absolute cell count. In addition, we found an association of the frequency of activated γδ T cell in the young at day 7 and Influenza A HAI titers at day 21.

## Materials and methods

### Study cohort

A total of 50 healthy donors at the age of 19–67 years were recruited into the study. The study was performed at Berlin—Brandenburg Center for Regenerative Therapies, Charité –Universitätsmedizin Berlin in Berlin in the fall of 2011 [37] and was approved by the ethics board of the Charité –Universitätsmedizin Berlin (approval numbers EA1/175/11). All study participants provided written informed consent before being recruited into the study. There were no children participating in the study. The inclusion criteria were no previous vaccination with any of the components in the 2011/2012 seasonal influenza vaccination to ensure a naïve immune status against 2011/2012 seasonal vaccination. Individuals who were receiving immunomodulatory therapy, had hemoglobin value less 12 g/dl, or were pregnant were excluded from the study. Donors who were suffering from an acute influenza like illness or other chronic illnesses prior to the study were likewise excluded, as were donors with a known allergy to any components of the vaccine. Donors who were sero-negative to at least one of the three strains in the influenza vaccine were included in this analysis. The resulting cohort was made of 21 young donors (19–30 years, 10 females, 11 males) and 23 elderly donors (53–67 years, 13 females, 10 males), summarized in Table 1.

**Table 1:** Cohort characteristics.

	Age group	
	Elderly	Young
Number donors	23	21
Age range (years)	53–67	19–30
Age mean (years)	58.65	26.14
Number female/male	13/10	10/11
Number CMV positive/negative	17/6	9/12
Number Day 0 sero-negative		
A/California/7/2009 (A(H1N1)pdm09)	20	17
A/Perth/16/2009 (A(H3N2))	19	17
B/Brisbane/60/2008	13	18

### Vaccination and sample collection

All donors received the trivalent inactivated influenza vaccine Mutagrip 2011/2012 (Sanofi-Pasteur) intramuscularly by a physician. The vaccine was composed of the following strains recommended by the WHO for the 2011/2012 influenza season in the northern hemisphere: an A/California/7/2009 (H1N1)-like virus, an A/Perth/16/2009 (H3N2)-like virus, a B/Brisbane/60/2008-like virus [38].

From each donor, a total of 50 ml of blood was drawn before injection of the vaccine and on day 3, 7, 10, 14, 17, and 21 after vaccination using Lithium-Heparin Vacutainers (BD Biosciences). The blood samples were processed immediately.

### Hemagglutination inhibition assay

Influenza specific serum antibody titers were measured by a standard hemagglutination inhibition (HAI) assay, using the seasonal 2011/2012 vaccine strains A/California/7/2009 (A(H1N1)pdm09), A/Perth/16/2009 (A(H3N2)), and B/Brisbane/60/2008 and erythrocytes from turkey hens as previously described [39]. Pre- and post (day 21) vaccination sera were tested simultaneously and in duplicates. Baseline sero-negativity was defined by a HAI titer below 10 [40].

### Preparation of PBMCs

Peripheral blood mononucleated cells (PBMCs) were prepared from whole blood by way of Leucosept-Tubes (Cellstar) per manufacturer’s instructions. Briefly, separation tubes were prepared with 15-ml Ficoll-Paque Plus (GE Healthcare) followed by addition of the heparin treated anticoagulated blood pre-diluted in PBS/BSA (Gibco) at a 1:1 ratio. Tubes were centrifuged at 800 g for 15 minutes at room temperature, and the PBMCs were isolated by gradient centrifugation and washed twice with PBS/BSA.

### Antibodies and staining procedure

Isolated PBMCs were stained with optimal dilutions of CD3-APC-H7 (Clone: SK7; BD Biosciences), γδTCR-APC (Clone: B1; BD Biosciences), CD38-PE (Clone: IB6; Miltenyi Biotec), Ki67-V450 (Clone: B56; BD Biosciences). Prior to staining with antibodies, unspecific antibody binding was blocked with Beriglobin (ZLB Behring) at a final concentration of 0.01 mg/ml. Surface staining was performed in PBS at 10^7^ cells/ml for 15 minutes at room temperature. Stained cells were fixed with the paraformaldehyde containing FACS-Lysing-Solution (BD Biosciences) for 10 minutes at room temperature in the dark. After permeabilization with FACS-Perm-Solution (BD Biosciences) for 10 minutes at room temperature, Ki67 was stained intra-cellular for 30 minutes at room temperature in the dark in FACS-Perm-Solution.

The cells were acquired on a MACS Quant (Miltenyi Biotec) flow cytometer. Quality control was performed daily using SPHERO Rainbow Calibration Particles (BD Biosciences). No adjustment to the PMT voltage was required between the runs. A compensation matrix was established the day before vaccination using single antibody stains.

### Data analysis

FACS data were analyzed using Flowjo version 9.8.3 (Tree Star) and statistical analysis was performed using R, version 3.2 [41]. Plots were generated with the R-package ggplot2 [42]. Values larger or smaller than 1.5 * *IRQ*, where IRQ is the inter-quartile range, were considered outliers. p-values < 0.05 after adjustment with false discovery rate (FDR) were considered significant [43]. Rate changes were determined by fitting the formula *y* = *β* + *αx* for linear kinetics and *y* = *β* + *α*/*x* for nonlinear kinetics.

## Results

**Fig 1:**
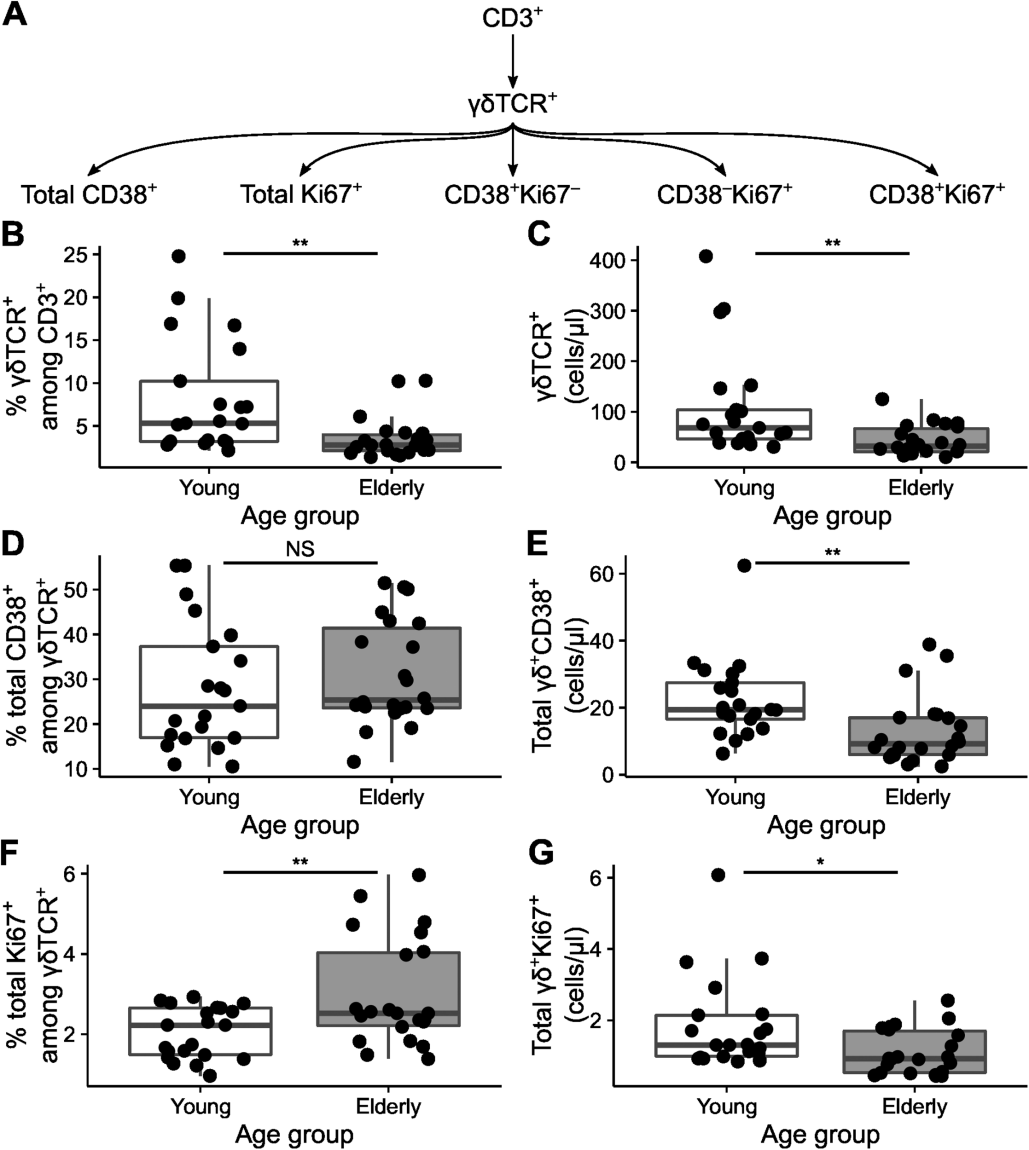
Baseline activation of γδ T cells changes with age. A) The gating hierarchy of the analyzed populations and their common CD3^+^ ancestor. Relative frequencies of γδTCR^+^ subsets (B) and their absolute counts per µl blood at day 0 are shown (C); total CD38^+^ among γδ T cells (D and E) cells, and total Ki67^+^ among γδ T cells (F and G). The box represents the 25^th^, 50^th^, and 75^th^ percentile and the whiskers represent the range of the observations excluding outliers. Each point signifies a single donor. NS indicate not significant, asterisks indicate p-values (** p < 0.01; * p < 0.05) after comparison with Student's t-test.

γδ T cells can become activated by influenza-specific proteins and present peptides derived from influenza virus particles to CD4^+^ and CD8^+^ αβ T cells [22,24]. The effect of aging on the kinetics of γδ T cells following vaccination with the trivalent 2011/2012 influenza vaccine was assessed in the PRIMAGE study cohort [37]. In this cohort healthy young (19–30 years of age) and elderly (53–67 years of age) donors were vaccinated with the seasonal 2011/2012 influenza vaccine, and the composition of circulating blood cells were analyzed by multi-parametric flow cytometry at day 0, 3, 7, 10, 14, 17, and 21. γδ T cells were identified by the γδTCR antibody clone B1. We focused on the detailed characterization of activated and proliferating γδ T cells as defined by the expression of CD38, Ki67, or combinations of CD38 and Ki67 (Fig 1A and Panel A in S1 Fig).

### Activation state of γδ T cells changes with age

We first looked at the overall proportion and absolute cell count of γδ T cells at baseline, before vaccination (Fig 1). We observed significantly lower frequency and absolute cell count of γδ T cells in the elderly compared to the young (Fig 1B and 1C). With respect to the activation and proliferative status (as defined by CD38 and Ki67 markers) we observed large interindividual variations. While no significant differences in the frequency of CD38^+^ among all γδ T cells were found between both age groups (Fig 1D), the absolute counts of γδ^+^CD38^+^ cells were significantly lower in elderly (Fig 1E), which reflect the overall decrease in γδ T cells detected in the elderly as compared to the young. In addition, the proportion of proliferating γδ T cells, as indicated by the expression of Ki67, were found to increase with age (Fig 1F), however due to overall decrease of γδ T cells the absolute count of total γδ^+^Ki67^+^ was decreased (Fig 1G). Taken together, our data demonstrate that at the baseline elderly showed a decreased number of γδ T cells and increased proportion of proliferating Ki67 γδ T as compared to the young.

### Heterogeneous response kinetics of cell frequencies

**Fig 2:**
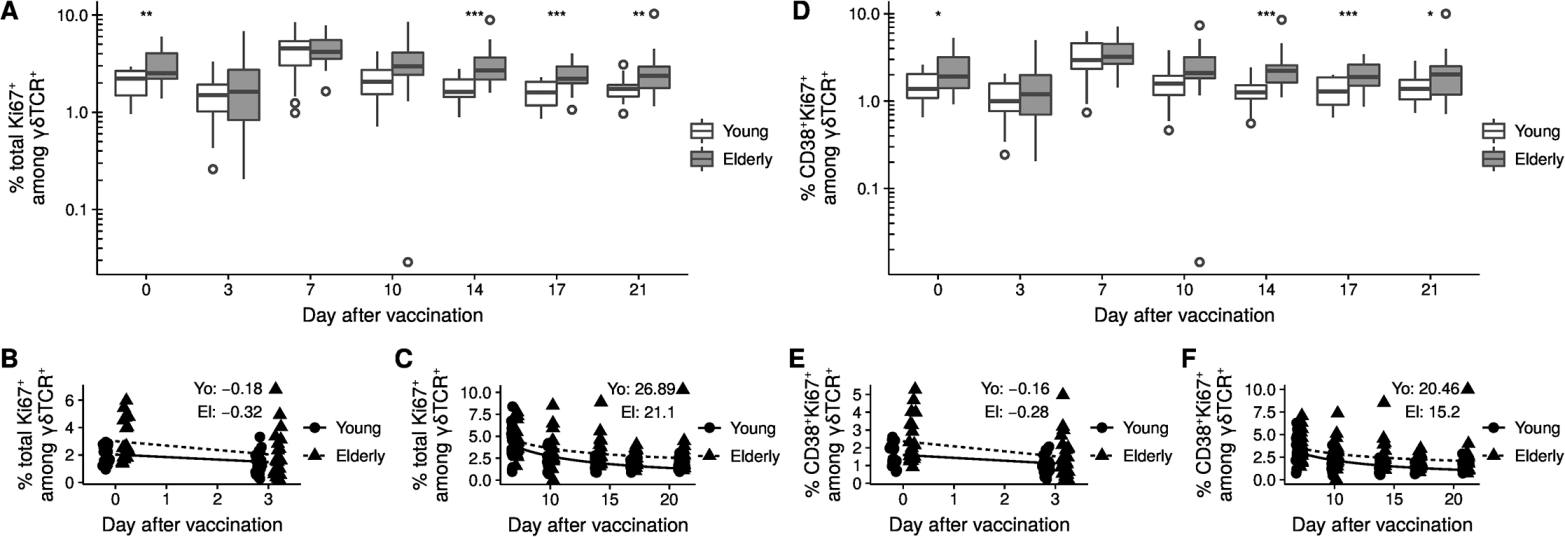
Activation status changes rapidly after vaccination. A) Total Ki67^+^ among γδTCR^+^. B) Proportionality in the segments day 0 to 3 and C) day 7 to day 21. D) CD38^+^Ki67^+^ among γδTCR^+^. E) Proportionality in the segments day 0 to 3, and F) day 7 to day 21. The box in figures A and D represents the 25^th^, 50^th^, and 75^th^ percentile and the whiskers the range excluding outliers (open circles). Asterisks indicate p-values (*** < 0.001; ** p < 0.01; * p < 0.05) after comparison with Student's t-test. p-values were corrected by the FDR method and only significant differences are shown. The two age groups in figures B, C, E, and F are slightly offset to the day of measurement to improve the visualization. Each point represents a single donor. The formula *y* = *β* + *αx* was fitted in B and E. The formula *y* = *β* + *α*/*x* was fitted in C and F. The given values indicate the proportionality constant α.

Next, we analyzed the kinetics of γδ T cells following vaccination. The decrease in the frequency of proliferating Ki67^+^ cells among CD3^+^γδTCR^+^ T cells from day 0 to day 3 was more pronounced in the elderly. Thus at day 3 the initial difference was abrogated (Fig 2A and 2B). Exemplary Ki67-stain is shown in Panel D in S1 Fig. The initial decrease was followed by a parallel increase to the similar peak values at day 7 in both groups. From this time point on, the frequency of proliferating Ki67^+^ cells among γδ T cells declined in both groups. The decrease was slower in the elderly and resulted in a significantly higher frequency of proliferating Ki67^+^ cells γδ T cells in elderly at day 14. The frequency of cells was stable from day 14 in the young and at day 17 for the elderly. The observed non-linear proportionality constants were 26.9 percent point (pp)*day in the young donors and 21.1 pp*day for the elderly individuals (Fig 2C).

Similar observations were made for the frequency of CD38^+^Ki67^+^ among γδTCR^+^ (Fig 2D–2F), but not for the cell count of these two populations (Panels A and B in S2 Fig). We further subdivided the age groups according to CMV status and observed similar kinetics (Panels A and B in S3 Fig). In summary, the data presented here demonstrate that the kinetics of proliferating γδ T cells differs between young and the elderly showing alterations in older individuals.

### Dynamic changes of activated γδ T cells with significantly lower levels in elderly

**Fig 3:**
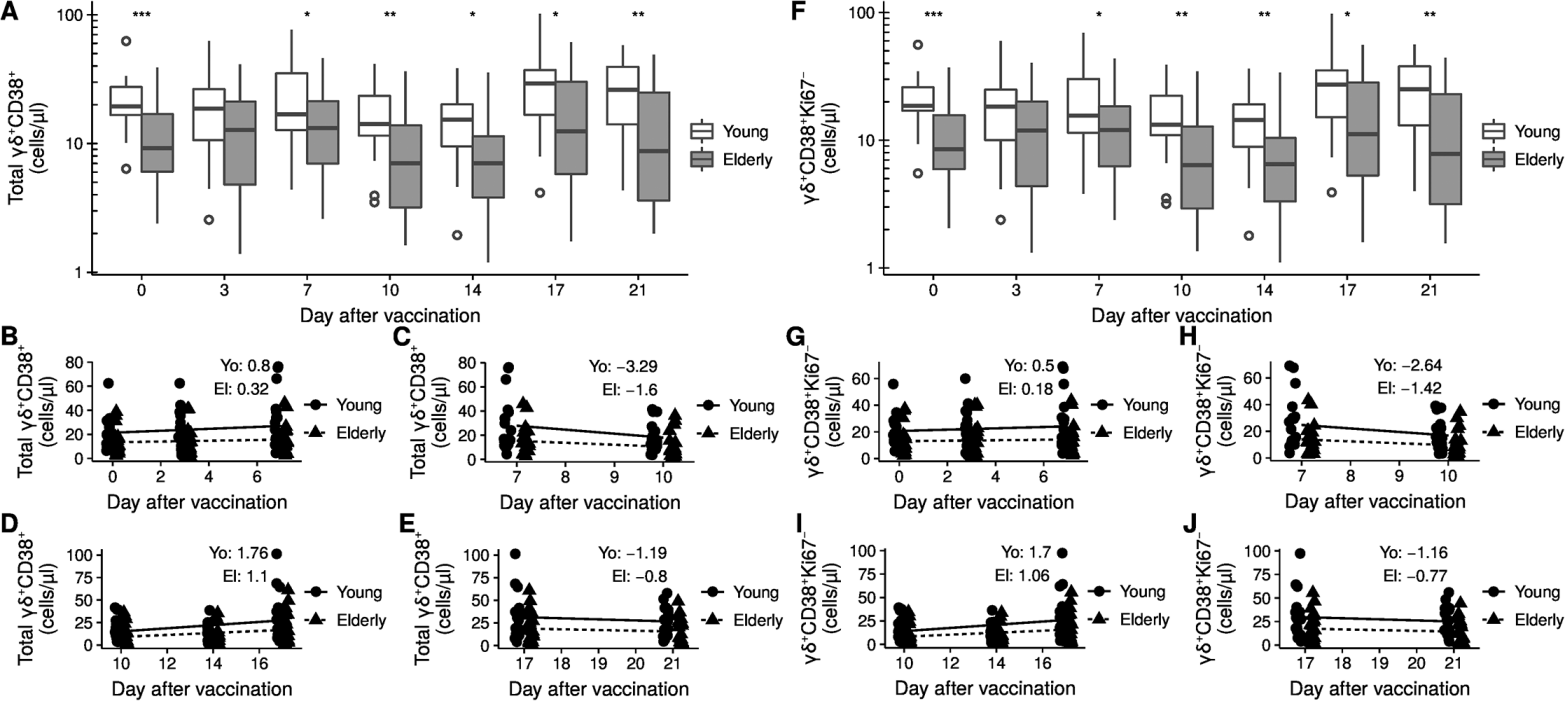
Dynamic changes of activated γδ T cells. A) Kinetics of absolute count of total CD38^+^ after vaccination. B) Linear fit for day 0 to 6, C) day 7 to 10, D) day 10 to 17, and E) day 17 to 21. F) Kinetics of absolute count of CD38^+^Ki67^–^ after vaccination. G) Linear fit for day 0 to 6, H) day 7 to 10, I) day 10 to 17, and J) day 17 to 21. The box in figures A and F represents the 25^th^, 50^th^, and 75^th^ percentile and the whiskers the range excluding outliers (open circles). Asterisks indicate p-values (*** < 0.001; ** p < 0.01; * p < 0.05) after comparison with Student's t-test. p-values were corrected by the FDR method and only significant differences are shown. The two age groups in figures B-E and in G-J are slightly offset to the day of measurement to improve the visualization. Each point represents a single donor. The formula *y* = *β* + *αx* was fitted in B-E and G-J. The given values indicate the proportionality constant α.

The absolute cell count of total CD38^+^ γδ T cells decreases with age in healthy individuals (Fig 1E). This difference in total CD38^+^γδTCR^+^ was abrogated at day 3 after vaccination but reinstated at day 7 (Fig 3A). In fact, the cell population revealed fluctuations among the young and the elderly alike on the days following vaccination. We therefore separated the time course into the following segments: day 0–7, day 7–10, day 10–17, and day 17–21. The increase during the first segment was stronger in the young with a rate of 0.8 cells/µl/day compared to 0.3 cells/µl/day for the elderly (Fig 3B). The subsequent decrease from day 7 to day 10 was twice as strong for the young with a rate of -3.3 cells/µl/day versus -1.6 cells/µl/day for the elderly (Fig 3C). In the following increase during the segment from day 10 to day 17 the young surpassed the elderly with rates of 1.8 cells/µl/day compared to 1.1 cells/µl/day for the elderly (Fig 3D). The decrease from day 17 to day 21 was similar for the two groups (Fig 3E).

A similar kinetics could be observed for the CD38^+^Ki67^–^ γδ T cells but not CD38^+^Ki67^+^γδTCR^+^ T cells (Fig 3F–3J and Panel B in S2 Fig). For the frequencies of total CD38^+^ and CD38^+^Ki67^–^ no difference in kinetics was observed (Fig 2C and Panel D in S2 Fig). When we divided the two age groups according to the CMV status, we observed kinetics similar to the complete age groups (Panels C and D in S3 Fig). Taken together, our data show that the kinetics of absolute count of activated γδ T cells is highly dynamic with larger changes in the young and significantly lower levels in elderly at all time points but day 3.

### Proliferation associates with vaccination titer

**Fig 4:**
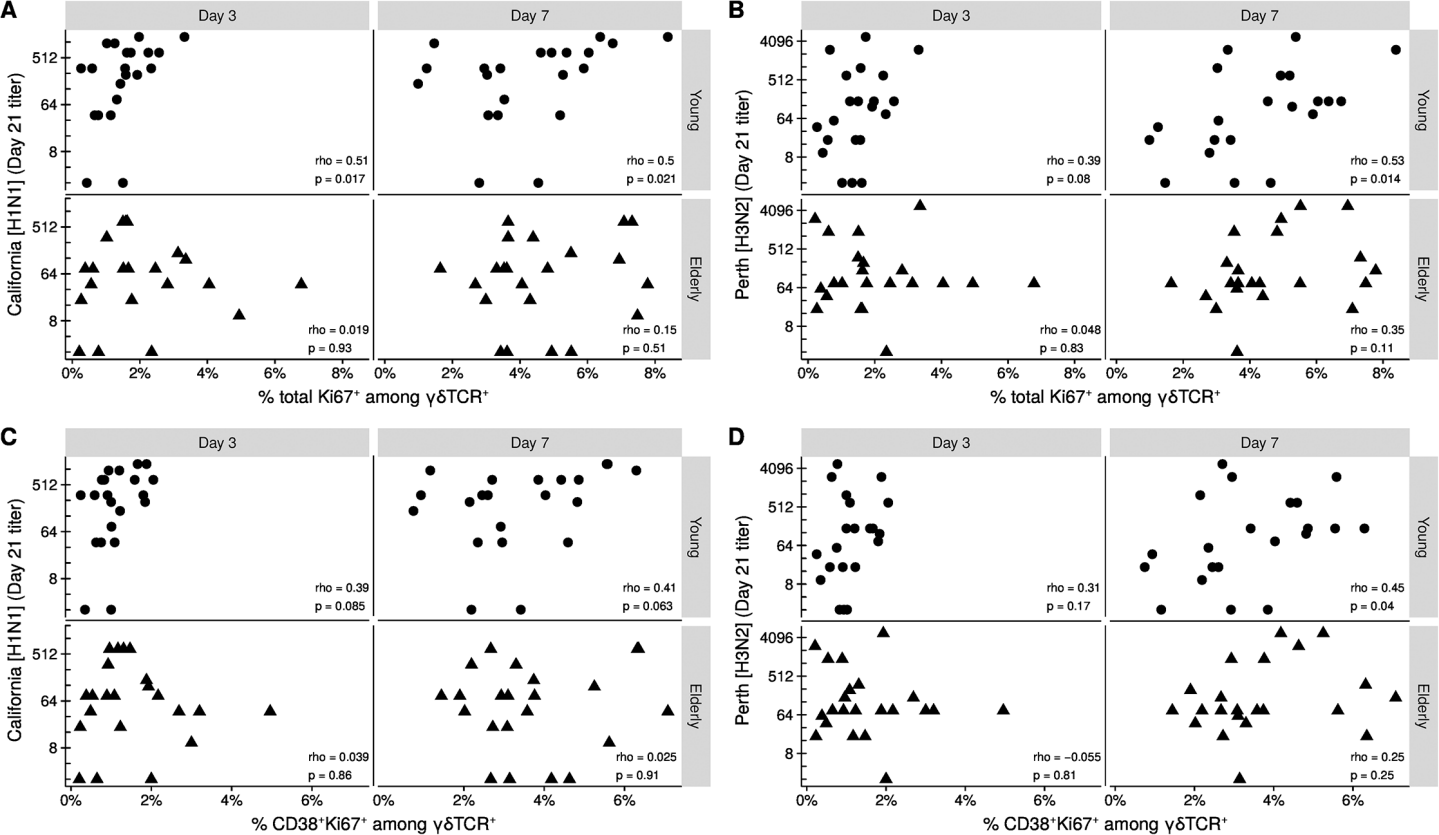
Proliferation level associates with vaccination titer in the young. Day 21 HAI titers of A/California/7/2009 (H1N1) and A/Perth/16/2009 (H3N2) compared to A-B) the frequency of total Ki67^+^ among γδTCR^+^ or C-D) the frequency of CD38^+^Ki67^+^ among γδTCR^+^ at day 3 and 7. Correlation by the Spearman rank method. Each point indicates a donor.

Next we compared the day 21 HAI titers for each strain in the vaccine to the frequency of proliferating γδ T cells (Fig 4 and S5 Fig). We found that the frequency of total Ki67^+^ cells among γδ T cells at day 3 associated with titers for A/California/7/2009 (H1N1) strain in the young (Fig 4A). Titers for both H1N1 and the other influenza A strain A/Perth/16/2009 (H3N2) associated with the frequency of total Ki67^+^ at day 7 in the young (Fig 4A and 4B). For the frequency of CD38^+^Ki67^+^ among γδTCR^+^ we only observed an association at day 7 in the young to H3N2 (Fig 4C and 4B). We did not observe any association between frequencies and the day 21 titers of the influenza B strain B/Brisbane/60/2008-like (S4 Fig). For the proportionality constants, only the rate change of proliferating cells from day 0 to day 3 associated with H1N1 titers in the young (S5 Fig). These data demonstrate a positive relationship between proliferating γδ T cells and vaccination outcome in the young but not in the elderly.

## Discussion

It is known that the adaptive immune system deteriorates with age in terms of diversity but also efficacy [44]. However, the effect of aging on γδ T cells especially in context of vaccination has not been investigated. Here we analyzed the kinetics of activated and proliferating γδ T cell subsets after seasonal influenza vaccination with respect to age. We further correlated the kinetics with the vaccination outcome. We show for the first time that the immediate γδ T cells response to pathogen, as well as the following kinetics, is altered with age. Our data also demonstrate an age dependent association between vaccination efficacy and proliferative capacity of γδ T cells.

The frequency of proliferating γδTCR^+^ cells was higher in the elderly at baseline. This increased proliferation rate might be a mechanism to counteract the overall decrease in γδ T cell numbers or reflect the low grade chronic inflammation generally observed in the elderly [45]. As a result, the γδ T cells in the elderly could potentially be exhausted. However, similar frequencies in the young and elderly were observed at the peak of the response. In addition, the functional state of exhaustion in γδ T cells is not clear [18].

The proportion of individuals infected with CMV increase with age as do the expansion of CMV specific αβ and γδ T cells [46,47]. This makes CMV infection a potential confounding factor in any study on age associated changes to T cell immunity. In addition, the γδ T cell subset Vδ2^–^ is expanded in CMV positive, while the Vδ2^+^ remained unchanged [48]. When we subdivided the age groups according to CMV status, we observed kinetics similar to those observed without subdivision, albeit not identical. This is probably due to loss of statistical power as the groups being compared become too small to reveal significant differences. The differences in kinetics presented in this report therefore do not depend on CMV status but stems from differences in age.

The CMV induced changes to the composition γδ T cell compartment in the elderly could explain the lack of association between activated cells and vaccination outcome in the elderly. However, in the elderly, we observe no difference in the frequencies of Ki67^+^ cells in the CMV negative and CMV positive sub-groups, indicating that the vaccination has the same effect in these two sub-groups.

Of interest, we found that proliferating cells are rapidly removed from the circulation in both the young and the elderly and reappear at the peak of response. Since γδ T cells can migrate to secondary lymphoid organs [25], it is possible that the disappearance of proliferating cells is caused by migration to the secondary lymphoid organs with a subsequent re-release into the circulation at a later time point. We found that the return to baseline levels occur at different rates depending on age, and it will be interesting to see mechanisms behind these differences and how this affects response to pathogens.

The frequency of Ki67^+^ cells among γδTCR^+^ cells in the young at day 3 and 7 associated with the HAI titer of the H1N1 like strain California, but only frequencies at day 7 associated with the other influenza A strain, the H3N2-like Perth. It is known that H3N2 and H1N1 can affect the immune response differently: while H3N2 might cause stronger symptoms [49,50], H1N1 might induce a more broadly neutralizing antibody response [51]. The observed difference in kinetics could potentially explain this difference in immune response.

Special attention was paid to the technical study performance. In this context, usage of reagents potentially affecting results was excluded. So, although the used γδ TCR antibody clone B1 is an activating antibody [52], the potential confounding effects in this study are negligible, as expression of Ki67 and CD38 remain stable for at least 4 h after activation [53,54]. It has also been reported that the B1 clone might not identify every γδ T cell when applied in a multicolor FACS panel [55]. However, we observed the expected range of γδ T cells of 1–10% among CD3^+^ in the young donors at baseline. The dynamic of the kinetics is therefore truthfully captured in our study. Limitations in our staining panel forced us to focus only on proliferating cells and cells with an activated phenotype. It will be interesting to see how age affects the kinetics of the major γδ T cells subpopulations.

In conclusion, γδ T cells in the peripheral blood respond rapidly to influenza vaccination irrespectively of age. Young individuals showed a more pronounced change in γδ T cell kinetics, while older individuals demonstrated overall lower cell counts of γδ T cells. Taken together, our study shows that early aging induces alterations of the γδ T cell response that might have implications on vaccination efficacy.

## Supporting information

S1 Fig Characterization of activation of γδ T cells. A) The gating strategy applied to identify activated γδTCR^+^ T cells. Shown is a representative donor. B and C) Activation status of γδTCR^+^ T cells at baseline in the young donors in Fig 1B separated into low (< 10%) and high (> = 10%) baseline frequency of γδTCR^+^ T cells. D) Representative Ki67 stain at day 0 and day 3 for an young and an elderly donor. The box in B and C represents the 25^th^, 50^th^, and 75^th^ percentile and the whiskers represent the range of the observations excluding outliers. Each point signifies a single donor. Asterisks indicate p-values (*** p < 0.001; ** p < 0.01) after comparison with Student's t-test.
(TIFF)

S2 Fig Kinetics of absolute counts and frequencies. Vaccination-dependent kinetics of absolute counts and frequencies of the γδ T cell subsets in Figs 2 and 3A) Absolute counts of γδTCR^+^Ki67^+^. B) Absolute counts of γδTCR^+^CD38^+^Ki67^+^. C) Frequency of total CD38^+^ among γδTCR^+^ T cells. D) Frequency of CD38^+^Ki67^–^ among γδTCR^+^ T cells. The box represents the 25^th^, 50^th^, and 75^th^ percentile and the whiskers represent the range of the observations excluding outliers (open circles). Asterisks indicate p-values (** p < 0.01; * p < 0.05) after comparison with Student's t-test. p-values were corrected by the FDR method and only significant differences are shown.
(TIFF)

S3 Fig CMV status and kinetics of absolute counts and frequencies. Vaccination-dependent kinetics of absolute counts and frequencies of the γδ T cell subsets in Figs 2 and 3, with age groups subdivided into CMV negative and CMV positive. A) Frequency of total Ki67^+^ among γδTCR^+^ T cells. B) Frequency of CD38^+^Ki67^+^ among γδTCR^+^ T cells. C) Absolute counts of γδTCR^+^CD38^+^. D) Absolute counts of γδTCR^+^CD38^+^Ki67^–^. The box represents the 25^th^, 50^th^, and 75^th^ percentile and the whiskers represent the range of the observations excluding outliers (open circles). Asterisks indicate p-values (** p < 0.01; * p < 0.05) after comparison of CMV negative young to CMV negative elderly, or CMV positive young to CMV positive elderly with Student's t-test. p-values were corrected by the FDR method and only significant differences are shown.
(TIFF)

S4 Fig Association of frequencies to day 21 HAI titers of B/Brisbane/60/2008. Association of day 21 HAI titers of B/Brisbane/60/2008-like virus to A) the frequency of total Ki67^+^ among γδTCR^+^ and B) the frequency of CD38^+^Ki67^+^ among γδTCR^+^ at day 3 and 7 for the young and the elderly. Correlation by the Spearman rank method. Each point indicates a donor.
(TIFF)

S5 Fig Association of proportionality constant and day 21 HAI titers. Association of day 0–3 proportionality constant of the frequency of total Ki67^+^ among γδTCR^+^ T cells and frequency of CD38^+^Ki67^+^ among γδTCR^+^ T cells for young and elderly (Fig 2B and 2E) to the day 21 HAI titers of the A) and D) A/California/7/2009 (H1N1), B) and E) A/Perth/16/2009 (H3N2), and C) and F) B/Brisbane/60/2008-like virus. Correlation by the Spearman rank method. Each point indicates a donor.
(TIFF)
