# Supplementary figures and images for: Age dependent differences in the kinetics of γδ T cells after influenza vaccination
Source: PLoS One. 2017 Jul 11;12(7):e0181161. doi: 10.1371/journal.pone.0181161 (PMC5507438; doi:10.1371/journal.pone.0181161)

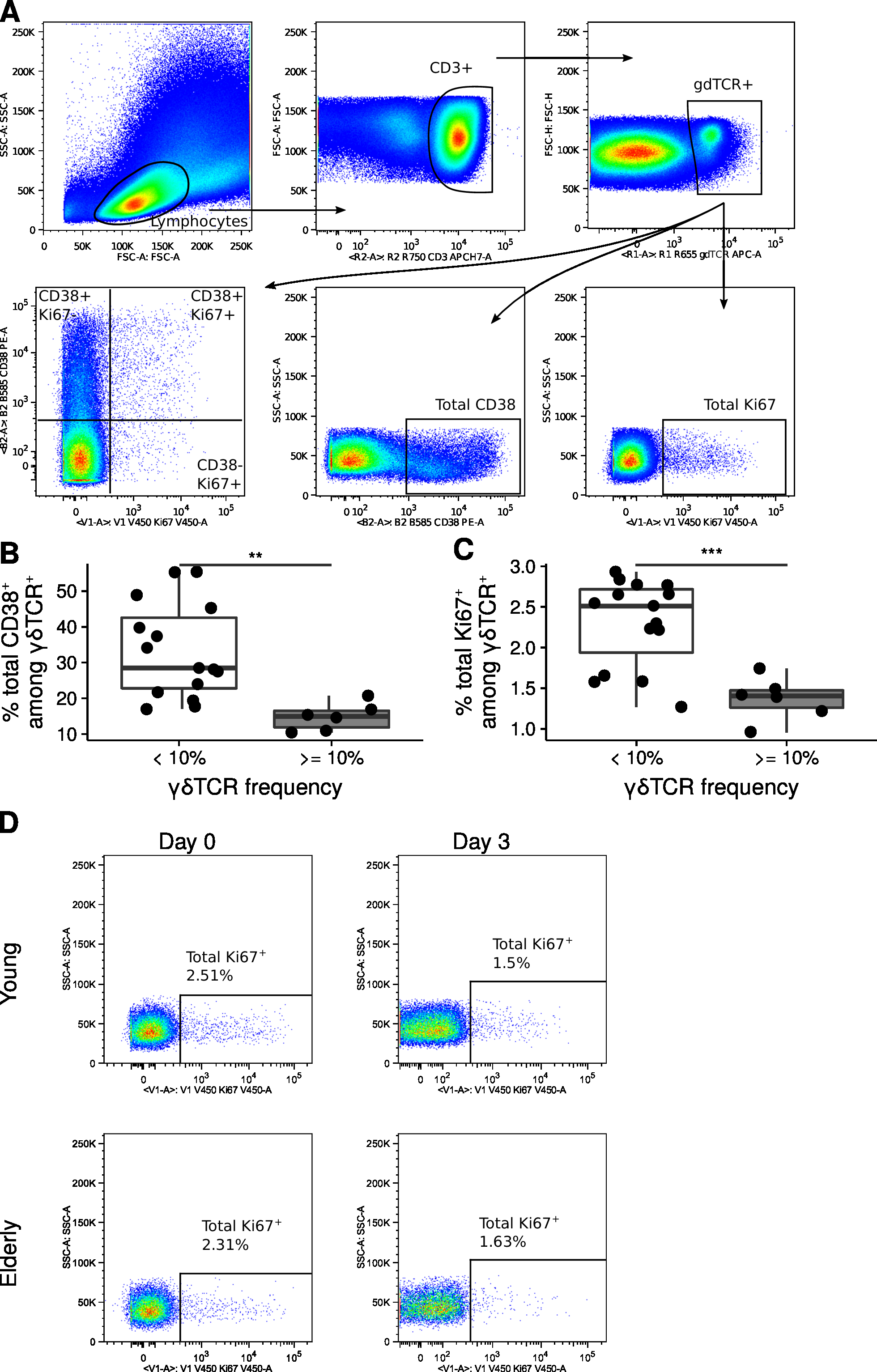

Supplement: S1 Fig — A) The gating strategy applied to identify activated γδTCR+ T cells. Shown is a representative donor. B and C) Activation status of γδTCR+ T cells at baseline in the young donors in Fig 1B separated into low (< 10%) and high (> = 10%) baseline frequency of γδTCR+ T cells. D) Representative Ki67 stain at day 0 and day 3 for an young and an elderly donor. The box in B and C represents the 25th, 50th, and 75th percentile and the whiskers represent the range of the observations excluding outliers. Each point signifies a single donor. Asterisks indicate p-values (*** p < 0.001; ** p < 0.01) after comparison with Student's t-test. (TIFF) [file pone.0181161.s001.tiff]

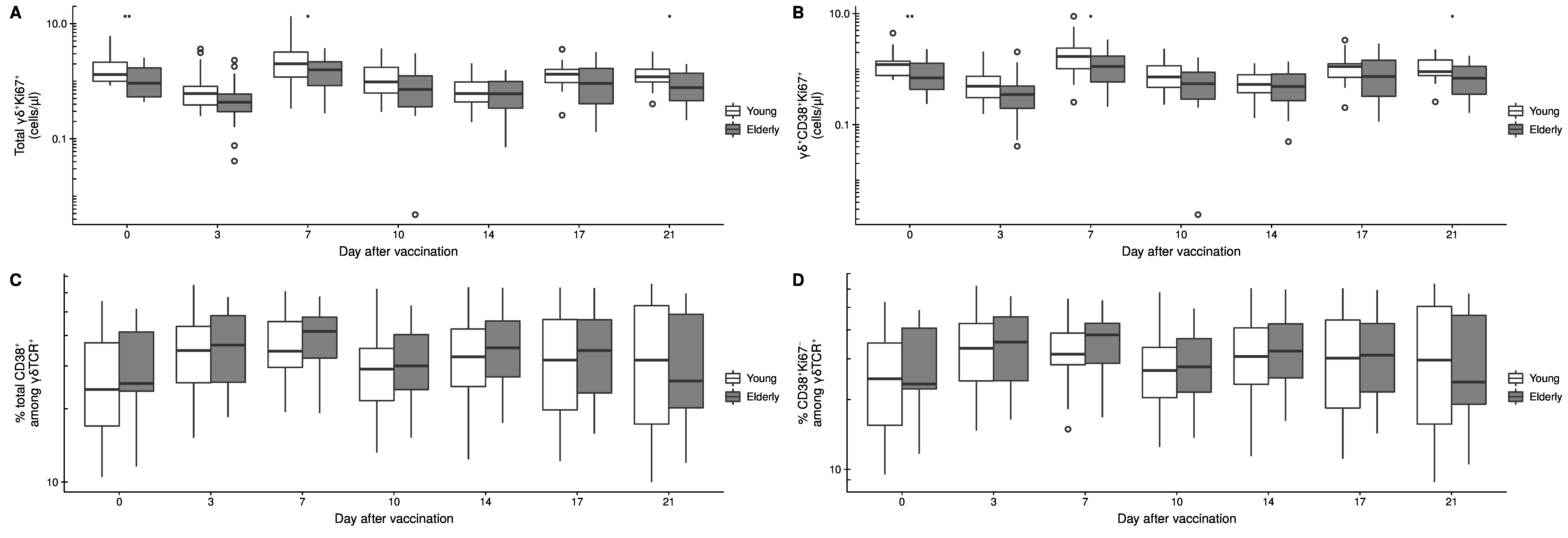

Supplement: S2 Fig — Vaccination-dependent kinetics of absolute counts and frequencies of the γδ T cell subsets in Figs 2 and 3A) Absolute counts of γδTCR+Ki67+. B) Absolute counts of γδTCR+CD38+Ki67+. C) Frequency of total CD38+ among γδTCR+ T cells. D) Frequency of CD38+Ki67– among γδTCR+ T cells. The box represents the 25th, 50th, and 75th percentile and the whiskers represent the range of the observations excluding outliers (open circles). Asterisks indicate p-values (** p < 0.01; * p < 0.05) after comparison with Student's t-test. p-values were corrected by the FDR method and only significant differences are shown. (TIFF) [file pone.0181161.s002.tiff]

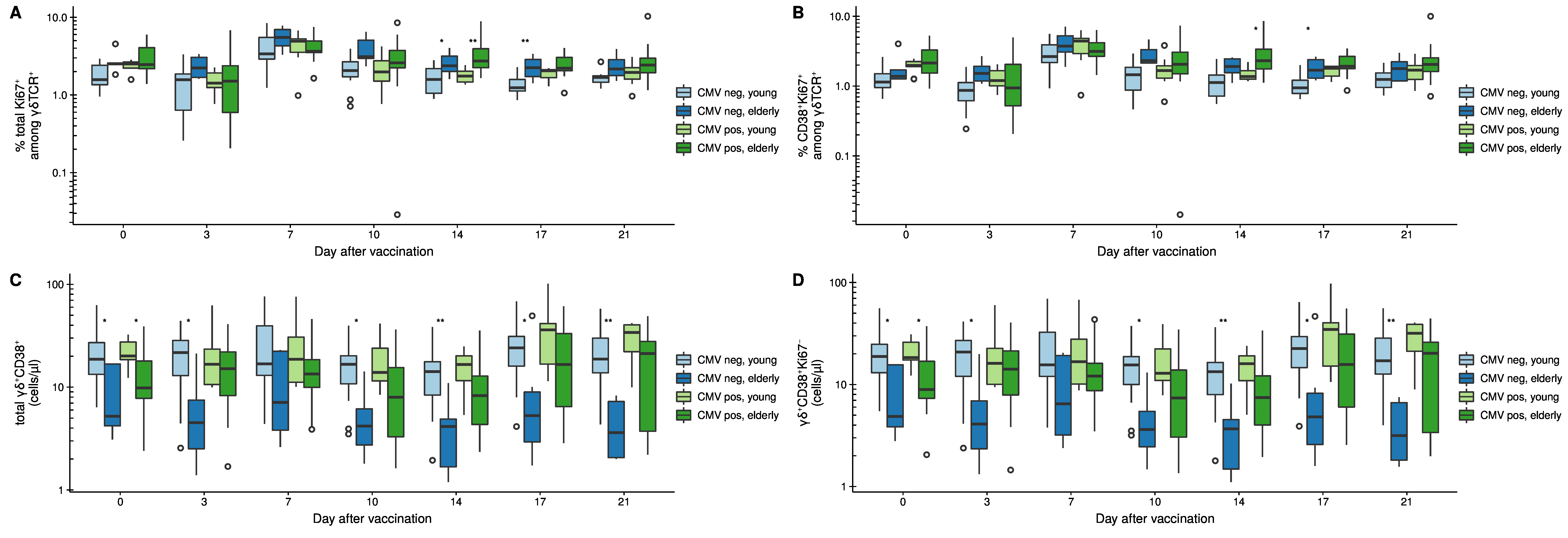

Supplement: S3 Fig — Vaccination-dependent kinetics of absolute counts and frequencies of the γδ T cell subsets in Figs 2 and 3, with age groups subdivided into CMV negative and CMV positive. A) Frequency of total Ki67+ among γδTCR+ T cells. B) Frequency of CD38+Ki67+ among γδTCR+ T cells. C) Absolute counts of γδTCR+CD38+. D) Absolute counts of γδTCR+CD38+Ki67–. The box represents the 25th, 50th, and 75th percentile and the whiskers represent the range of the observations excluding outliers (open circles). Asterisks indicate p-values (** p < 0.01; * p < 0.05) after comparison of CMV negative young to CMV negative elderly, or CMV positive young to CMV positive elderly with Student's t-test. p-values were corrected by the FDR method and only significant differences are shown. (TIFF) [file pone.0181161.s003.tiff]

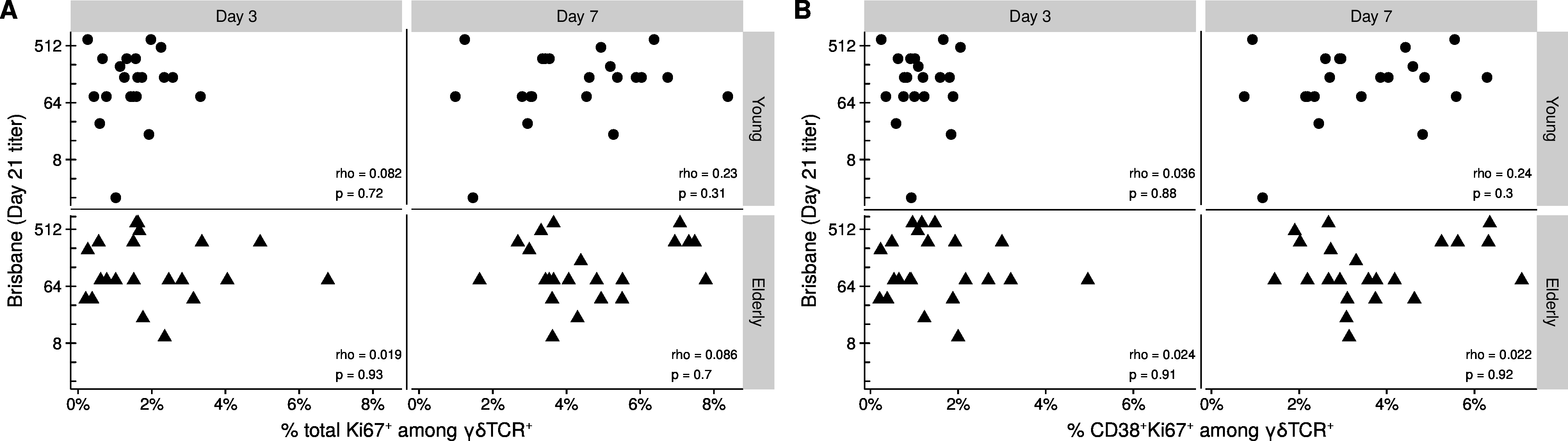

Supplement: S4 Fig — Association of day 21 HAI titers of B/Brisbane/60/2008-like virus to A) the frequency of total Ki67+ among γδTCR+ and B) the frequency of CD38+Ki67+ among γδTCR+ at day 3 and 7 for the young and the elderly. Correlation by the Spearman rank method. Each point indicates a donor. (TIFF) [file pone.0181161.s004.tiff]

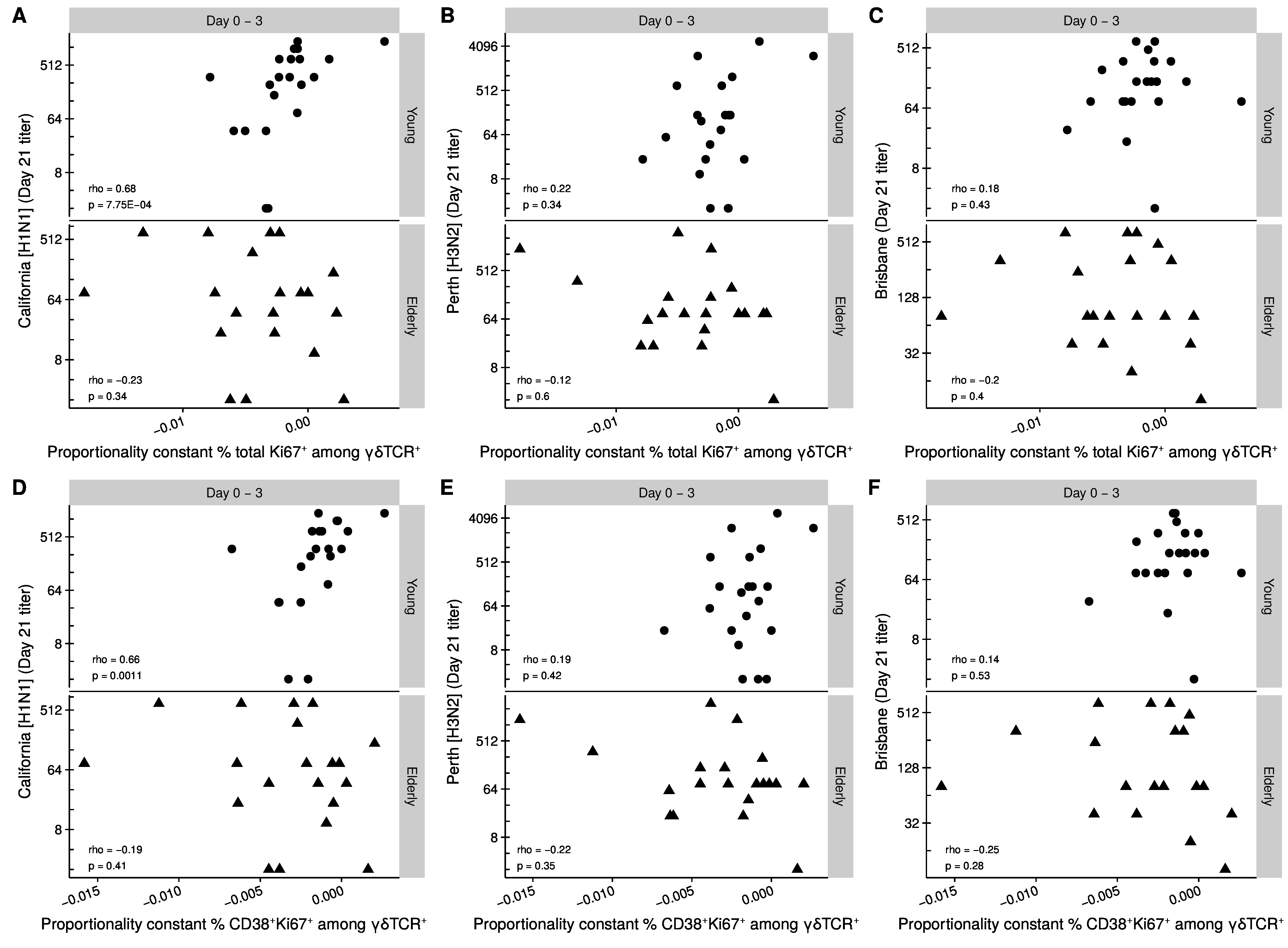

Supplement: S5 Fig — Association of day 0–3 proportionality constant of the frequency of total Ki67+ among γδTCR+ T cells and frequency of CD38+Ki67+ among γδTCR+ T cells for young and elderly (Fig 2B and 2E) to the day 21 HAI titers of the A) and D) A/California/7/2009 (H1N1), B) and E) A/Perth/16/2009 (H3N2), and C) and F) B/Brisbane/60/2008-like virus. Correlation by the Spearman rank method. Each point indicates a donor. (TIFF) [file pone.0181161.s005.tiff]
